# Indole Alkaloids from Marine Sources as Potential Leads against Infectious Diseases

**DOI:** 10.1155/2014/375423

**Published:** 2014-06-05

**Authors:** Paulo H. B. França, Daniel P. Barbosa, Daniel L. da Silva, Êurica A. N. Ribeiro, Antônio E. G. Santana, Bárbara V. O. Santos, José M. Barbosa-Filho, Jullyana S. S. Quintans, Rosana S. S. Barreto, Lucindo J. Quintans-Júnior, João X. de Araújo-Júnior

**Affiliations:** ^1^Institute of Chemistry and Biotechnology, Federal University of Alagoas, University City, BR 101, KM 14 Norte, Tabuleiro dos Martins, 57072-970 Maceio, AL, Brazil; ^2^School of Nursing and Pharmacy, Federal University of Alagoas, Av. Lourival de Mello Motta, S/N, Tabuleiro dos Martins, 57072-970 Maceió, AL, Brazil; ^3^Laboratory of Pharmaceutical Technology, Federal University of Paraiba, 58051-900 João Pessoa, PB, Brazil; ^4^Department of Physiology, Federal University of Sergipe, 49000-100 São Cristóvão, SE, Brazil

## Abstract

Indole alkaloids comprise a large and complex class of natural products found in a variety of marine sources. Infectious diseases remain a major threat to public health, and in the absence of long-term protective vaccines, the control of these infectious diseases is based on a small number of chemotherapeutic agents. Furthermore, the emerging resistance against these drugs makes it urgently necessary to discover and develop new, safe and, effective anti-infective agents. In this regard, the aim of this review is to highlight indole alkaloids from marine sources which have been shown to demonstrate activity against infectious diseases.

## 1. Introduction

Seas and oceans occupy more than 75% of the Earth's surface and contain nearly all groups of organisms, including representatives of 34 out of 36 phyla described. Thus, marine ecosystems can be considered as having the greatest phyletic biodiversity with virtually unlimited biotechnological potential [1]. The marine environment is massively complex, consisting of extreme variations in pressure, salinity, temperature, and biological habitats that have led to the production of several novel structures with unique biological properties, which may not be found in terrestrial natural products [2]. In the past 30–40 years, marine plants and animals have been the focus of a worldwide effort to define the natural products of the marine environment. A small number of marine plants, animals, and microorganisms have already yielded more than 12,000 novel chemicals, with hundreds of new compounds still being discovered every year. These discovery efforts have yielded several bioactive metabolites that have been successfully developed by the pharmaceutical industry [3]. A variety of marine sources including sponges, tunicates, red algae, acorn worms, and symbiotic bacteria have been shown to generate indole alkaloids, which represent the largest number and most complicated of the marine alkaloids [4–7]. The alkaloids obtained from marine organisms frequently possess novel frameworks which cannot be found in terrestrially related organisms. Marine metabolites often possess complexities such as halogen substituents [8]. In addition, bearing in mind that several species of organisms are associated with cyanobacteria and bacteria, it is considered that several natural products originating in microorganisms can be isolated from marine animals [9]. Indole alkaloids have been shown to exhibit a wide array of biological activities such as opioid receptor agonistic [10], antibacterial [11, 12], antifungal [13, 14], anti-inflammatory [15], antileishmanial [16, 17], antiplasmodial [18, 19], anti-HIV [20], cytotoxic [21], glucose uptake stimulatory [22], larvicidal [23], trypanocidal [24], and vasodilator [25, 26] and inhibition of cholinesterase [27], indoleamine-2, 3-dioxygenase [28], calmodulin [29], and CB1 cannabinoid receptor [30]. Infectious diseases caused by bacteria, fungi, viruses, and parasites are still a major threat to public health, despite the tremendous progress in human medicine. The high prevalence of these diseases and the emergence of widespread drug resistance developed by these parasites to current treatments, leading to reduction of their efficacy and consequent increase in the cost of conventional treatments, highlight the need for novel and effective therapeutic alternatives with fewer or no side-effects. Their impact is particularly large in developing countries due to the relative unavailability of medicines [31, 32].

Therefore, in continuation of our research on bioactive molecules from natural origin [33–53] and plant extracts [54–63] we offer this compilation of the indole alkaloids from marine sources.

In this review, literature was covered in order to highlight alkaloidal compounds with an indole moiety which have been shown to demonstrate activity against infectious diseases.

## 2. Indole Alkaloids from Marine Sources

**Figure 1:**
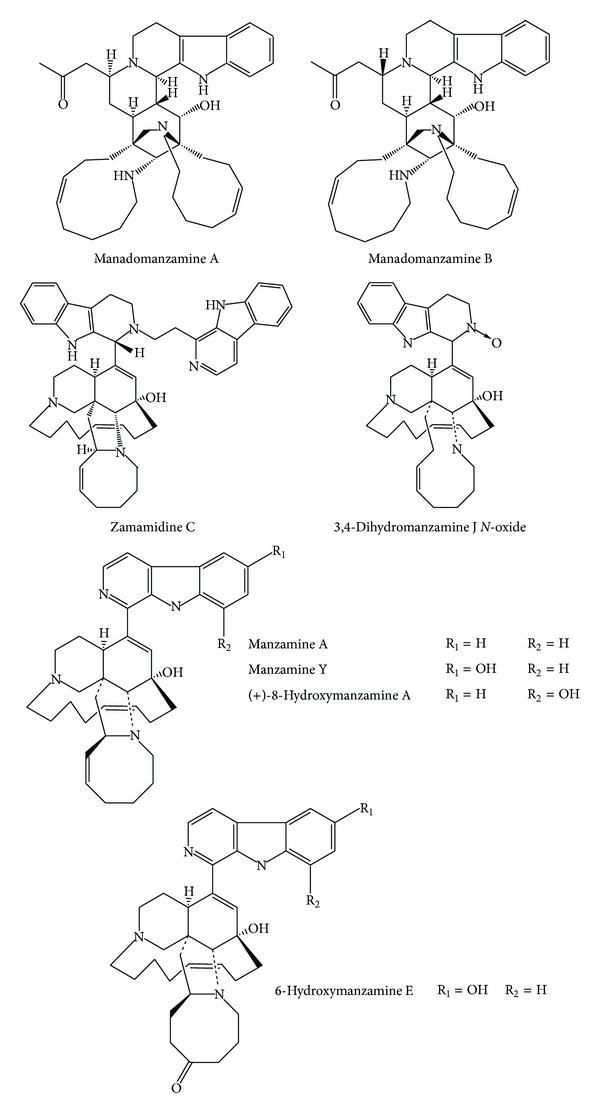
Structures of *β*-carbolines with manzamine-type frameworks.

Manadomanzamines A and B (Figure 1), *β*-carbolines with a novel rearrangement of the manzamine framework, were isolated from an Indonesian sponge* Acanthostrongylophora* sp. These compounds showed significant activities against* Mycobacterium tuberculosis*, with MIC values of 1.9 and 1.5 *μ*g/mL, respectively. Manadomanzamines A and B were also active against human immunodeficiency virus (HIV-1) with EC_50_ values of 7.0 and 16.5 *μ*g/mL. Furthermore, manadomanzamine A was active against the fungus* Candida albicans* with an IC_50_ of 20 *μ*g/mL, whilst manadomanzamine B exhibited activity against the fungus* Cryptococcus neoformans* with IC_50_ value of 3.5 *μ*g/mL [64].

Manzamine A, a *β*-carboline alkaloid present in several marine sponge species, has been shown to inhibit the growth of the rodent malaria parasite* Plasmodium berghei* in vivo. More than 90% of the asexual erythrocytic stages of* P. berghei* were inhibited after a single intraperitoneal injection of manzamine A into infected mice. Moreover, it was demonstrated that immunostimulatory effects caused by the compound play an important role in preventing mouse death due to fulminating recurrent parasitemia in animals treated with 100 mmol per kg of manzamine A [65].

Several manzamine-type alkaloids were also isolated from an Indonesian sponge of the genus* Acanthostrongylophora.* The *β*-carbolines (+)-8-hydroxymanzamine A and 6-hydroxymanzamine E (Figure 1) showed strong activity against* M. tuberculosis* H37Rv with MIC values of 0.9 and 0.4 *μ*g/mL, respectively. Manzamine A and (+)-8-hydroxymanzamine were strongly active when tested against* Plasmodium falciparum* chloroquine-sensitive D6 clone and chloroquine-resistant W2 clone, showing, respectively, IC_50_ values of 4.5 and 6.0 ng/mL toward the former and equally 8.0 ng/mL toward the latter. Moreover, manzamine A, manzamine Y, and 6-hydroxymanzamine E (Figure 1) have shown significant activity against* Leishmania donovani*, with IC_50_ values of 0.9, 1.6, and 2.5 *μ*g/mL, respectively [66].

Investigation of bioactivity of manzamine alkaloids isolated from an Okinawan sponge* Amphimedon* sp. revealed active compounds against* Trypanosoma brucei brucei* and* P. falciparum. *Zamamidine C (Figure 1), 3,4-dihydromanzamine J* N*-oxide, and manzamine A showed inhibitory activities* in vitro* against* T. b. brucei* with IC_50_ values of 0.27, 4.44, and 0.04 *μ*g/mL, respectively, and* P. falciparum* with IC_50_ values 0.58, 7.02, and 0.97 *μ*g/mL, respectively [67].

**Figure 2:**
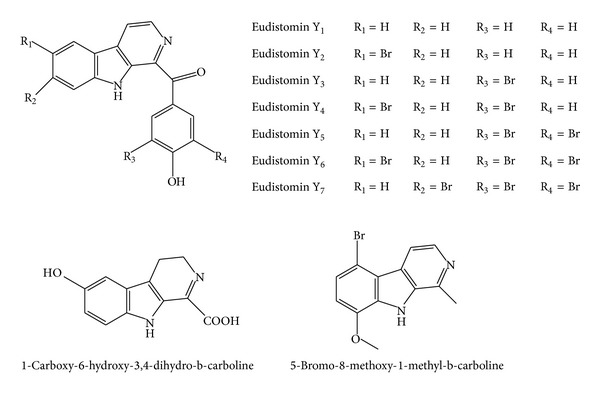
Structures of *β*-carbolines with eudistomin-derived scaffold and simple *β*-carbolines.

Seven *β*-carboline-based metabolites, designated as eudistomins Y_1_–Y_7_ (Figure 2), were isolated from a tunicate of the genus* Eudistoma* collected in South Korea. These new metabolites differ from previously isolated marine metabolites due to the presence of a benzoyl group attached to the *β*-carboline nucleus at C-1. Eudistomin Y_6_ exhibited moderate antibacterial activity against Gram-positive bacteria* Staphylococcus epidermidis* ATCC12228 and* B. subtilis* ATCC 6633 with MIC values of 12.5 and 25 *μ*g/mL, respectively, but showed no inhibitory activity toward the other two strains of Gram-positive bacteria,* S. aureus* ATCC 6538 and* M. lutes* ATCC 9341, and the Gram-negative bacteria including* E. coli* ATCC 11775,* Salmonella typhimurium *ATCC 14028, and* Klebsiella pneumoniae* ATCC 4352. Eudistomins Y_1_ and Y_4_ also displayed the same selectivity as eudistomin Y_6_ but demonstrated weak antibacterial activity against the two strains of bacteria* S. epidermidis* ATCC12228 and* B. subtilis* ATCC 6633 with MICs of 50 and 200 *μ*g/mL, respectively [68].

Investigation of the CH_2_Cl_2_ extract from the bryozoan* Pterocella vesiculosa*, collected in New Zealand, has led to the isolation of 5-bromo-8-methoxy-1-methyl-*β*-carboline (Figure 2). This alkaloid was evaluated for antibacterial and antifungal activities and showed inhibitory action toward the Gram-positive bacterium* B. subtilis* and the fungi* C. albicans* and* Trichophyton mentagrophytes* with MID ranges of 2–4, 4-5 and 4-5 *μ*g/mL, respectively [69].

**Figure 3:**
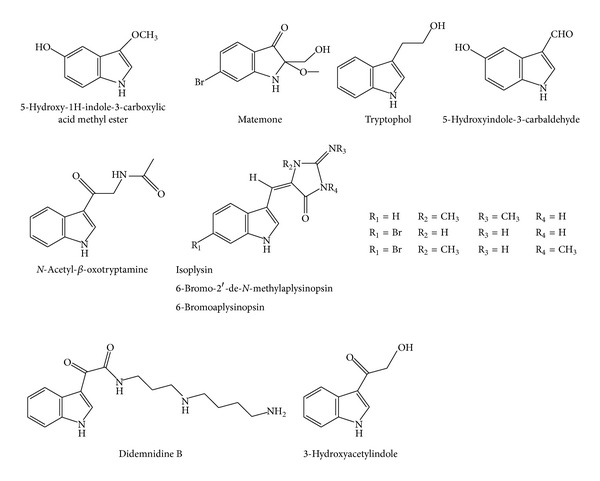
Structures of simple indole alkaloids.

An indole spermidine alkaloid, didemnidine B (Figure 3), was described in the New Zealand ascidian* Didemnum* sp. Evaluation of the compound against* Trypanosoma brucei rhodesiense*,* Trypanosoma cruzi*,* L. donovani*, and* Plasmodium falciparum* K1 chloroquine-resistant strain indicated didemnidine B to be mildly active toward the malaria parasite with IC_50_ value of 15 *μ*M [70].

**Figure 4:**
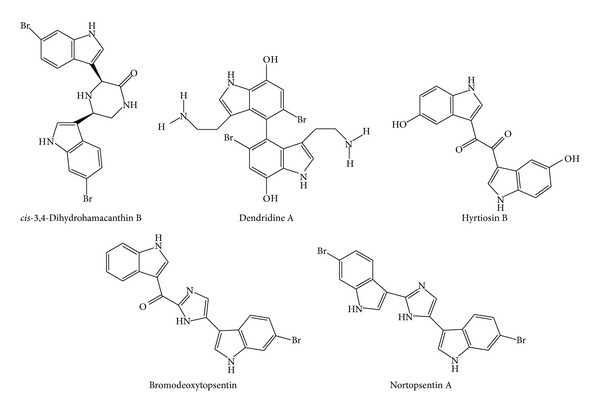
Structures of bis-indole alkaloids.

In addition, chemical investigations of the tropical marine sponge* Hyrtios* sp. have resulted in the isolation of several alkaloids, which were evaluated as* C. albicans* isocitrate lyase inhibitors. Out of the compounds tested, the bis-indole alkaloid hyrtiosin B (Figure 4) showed the most potent inhibitory activity with an IC_50_ value of 50.7 *μ*M. Other compounds comprised simple indole alkaloids, namely, 1-carboxy-6-hydroxy-3,4-dihydro-*β*-carboline, 5-hydroxy-1*H*-indole-3-carboxylic acid methyl ester, serotonin, hyrtiosin A, and 5-hydroxyindole-3-carbaldehyde (Figure 3) revealed only moderate to weak activity against* C. albicans* isocitrate lyase, with MIC values ranging between 39.8 and 152.9 *μ*M. By comparing their chemical structures, it was found that the enzyme inhibitory activities of these 5-hydroxyindole-type alkaloids are markedly affected by a substitution of functional group at the C-3 position. A substitution by a hydrophilic group at the C-3 position results in an increase of the isocitrate lyase inhibitory activity [71].

Tryptophol (Figure 3), a simple indole alkaloid from sponge* Ircinia spinulosa*, was screened for antitrypanosomal activity. The compound was active against* T. b. rhodesiense* with an IC_50_ value of 5.89 *μ*g/mL, but it was 8-fold less active against* T. cruzi*, with IC_50_ value of 49.37 *μ*g/mL, than against* T. b. rhodesiense*. Toward* L. donovani*, tryptophol displayed an IC_50_ value of 9.60 *μ*g/mL, compared with 0.20 *μ*g/mL of the standard drug, miltefosine. For* P. falciparum*, it was shown that tryptophol presented an IC_50_ value of 5.08 *μ*g/mL, compared with 0.056 *μ*g/mL of chloroquine [72].

A bioactive bromine-containing oxindole alkaloid, matemone (Figure 3), was isolated from the Indian Ocean sponge* Iotrochota purpurea*. Matemone showed marginal antimicrobial activity against the bacterium* Staphylococcus aureus* at 50, 100, and 200 *μ*g/disk, affording inhibitory zones of 7, 9, and 11 mm, respectively [73].

Dendridine A (Figure 4), a C2-symmetrical 4,4′-bis(7-hydroxy) indole alkaloid, was reported in extracts of an Okinawan sponge* Dictyodendrilla* sp. Dendridine A exhibited inhibitory activities against Gram-positive bacteria* Bacillus subtilis* and* Micrococcus luteus* with MIC values of 8.3 and 4.2 *μ*g/mL, respectively, and the fungus* C. neoformans* with MIC of 8.3 *μ*g/mL [74].

In order to discover active compounds with inhibitory activity against methicillin-resistant* S. aureus* pyruvate kinase (MRSA-PK), screening of an extract library of marine invertebrates resulted in the identification of bis-indole alkaloids from the* Topsentia pachastrelloides*. The most active compounds,* cis*-3,4-dihydrohamacanthin B and bromodeoxytopsentin (Figure 4), were identified as highly potent MRSA-PK inhibitors with IC_50_ values of 16–60 nM and with at least 166-fold selectivity over human PK isoforms. These novel anti-PK natural compounds exhibited significant antibacterial activities against MRSA with MIC values of 12.5 and 6.25 *μ*g/mL, respectively, and selectivity indices (CC_50_/MIC) > 4 [75].

The bis-indole alkaloid nortopsentin A (Figure 4) present in enriched fractions of marine sponges from genus* Spongosorites* exhibited potent inhibition of* Plasmodium falciparum* growth. Assays were performed in chloroquine-sensitive (3D7) and chloroquine-resistant (Dd2) strains, and IC_50_ values obtained were 460 nM against the former and 560 nM against the latter [76].

The bisindole alkaloid (*R*)-6′-debromohamacanthin B (Figure 4) was isolated from the MeOH extract of a marine sponge* Spongosorites* sp. and showed weak antibacterial activity against clinically isolated methicillin-resistant strains, with MIC values of 6.3 *μ*g/mL for* S. pyogenes* 308A and 12.5 *μ*g/mL for* S. pyogenes* 77A,* S. aureus* SG 511;* S. aureus* 285;* S. aureus* 503 [77].

Bis-indole alkaloids isolated from the Jamaican sponge* Smenospongia aurea *were tested against the D6 clone of* P. falciparum* for their* in vitro* antimalarial activity. The compound 6-bromoaplysinopsin (Figure 4) exhibited activity at endpoints of 0.087 and 0.34 *μ*g/mL with a selectivity index of 55 and 14, respectively. Other compounds such as isoplysin A and 6-bromo-2′-de-*N*-methylaplysinopsin (Figure 3) showed moderate activity at 0.97, 1.1, and 0.94 *μ*g/mL with a selectivity index of >4.9, >4.3, and >5.1, respectively. Moreover, 6-bromo-2′-de-*N*-methylaplysinopsin inhibited the antimalarial target plasmepsin II enzyme with IC_50_ 53 *μ*M in fluorescence resonance energy transfer (FRET) and 66 *μ*M in fluorescence polarization (FP) assays [78].

**Figure 5:**
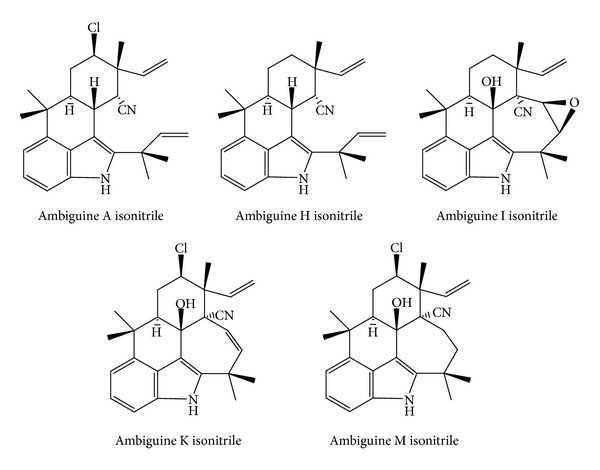
Structures of ambiguine isonitriles.

Bioassay-guided fractionation of the 7 : 3 MeOH/water extract of a cultured cyanobacterium strain identified as* Fischerella* sp. yielded nine isonitrile-containing alkaloids. Ambiguine H isonitrile and ambiguine I (Figure 5) isonitrile exhibited strong activity against the bacteria* Staphylococcus albus *(MIC values of 0.625 and 0.078 *μ*g/mL) and* B. subtilis* (MIC values of 1.25 and 0.312 *μ*g/mL). Ambiguine I isonitrile was strongly active against the fungi* Saccharomyces cerevisiae* (MIC value of 0.312 *μ*g/mL) and* C. albicans* ATCC 90028 (MIC value of 0.39 *μ*g/mL) [79].

A new investigation on ambiguines highlighted that ambiguine A isonitrile showed MIC of 1.0 *μ*M against* B. anthracis*, while ambiguine K and M isonitriles (Figure 5) were more potent towards* M. tuberculosis* with MIC values of 6.6 *μ*M and 7.5 *μ*M, respectively [80].

**Figure 6:**
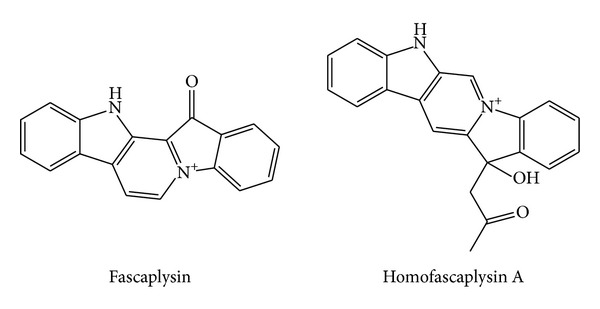
Structures of fascaplysin and homofascaplysin A.

From the CH_2_Cl_2_ extract of the sponge* Hyrtios* cf.* erecta*, collected in Fiji, *β*-carbolines homofascaplysin A and fascaplysin (Figure 6) were isolated. Evaluation of the biological activity of the compounds toward* P. falciparum* revealed that homofascaplysin and fascaplysin are both potently active* in vitro* against the parasite. Homofascaplysin A also inhibited the growth of* Escherichia coli *(50 *μ*g/9 mm) and* Bacillus megaterium *(50 *μ*g/11 mm). Fascaplysin inhibited the growth of* E. coli* (50 *μ*g/6 mm) and* B. megaterium* (50 *μ*g/10 mm). Further biological activity for fascaplysin was found against* Trypanosoma b. rhodesiense*, displaying moderate activity with IC_50_ value of 0.17 *μ*g/mL compared with melarsoprol, which showed IC_50_ value of 2 ng/mL. The evaluation of antiviral activity of fascaplysin revealed an increased cytopathogenic effect at noncytotoxic concentrations of 0.038 *μ*g/mL toward fetal Rhesus monkey kidney cells (FRhK-4-cells) infected with the HAV-variant HAVcytHB1.1. Persistently infected FRhK-4-cells (HAV/7) showed cytopathogenicity at 0.038 *μ*g/mL of fascaplysin, while untreated FRhK-4-cells remained unchanged. Homofascaplysin A and fascaplysin were shown to be potent* in vitro* inhibitors of chloroquine-susceptible (NF54) and chloroquine-resistant* P. falciparum *strains. Positive control substances were chloroquine and artemisinin. The potency against the K1 strain of homofascaplysin A was stronger than that of chloroquine. Compared with artemisinin (K1 strain) and with both positive control substances (NF54 strain), homofascaplysin was approximately 10-fold less active [81].

**Figure 7:**
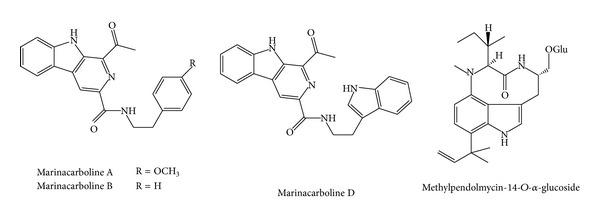
Structures of marinacarbolines and pendolmycin derivative.

Alkaloids obtained from the fermentation broth of* Marinactinospora thermotolerans* SCSIO 0652 were tested for their antiplasmodial activities against* P. falciparum* line 3D7, a drug-sensitive strain, and Dd2, a multi-drug-resistant strain. The results of the antiplasmodial assays revealed that marinacarboline A (Figure 7) and methylpendolmycin-14-*O*-**α**-glucoside (Figure 6) inhibited* P. falciparum* line Dd2 with IC_50_ values of 1.92 and 5.03 *μ*M, respectively, and marinacarbolines C and D inhibited* P. falciparum* lines 3D7 and Dd2 with IC_50_ values between 3.09 and 5.39 *μ*M [82].

**Figure 8:**
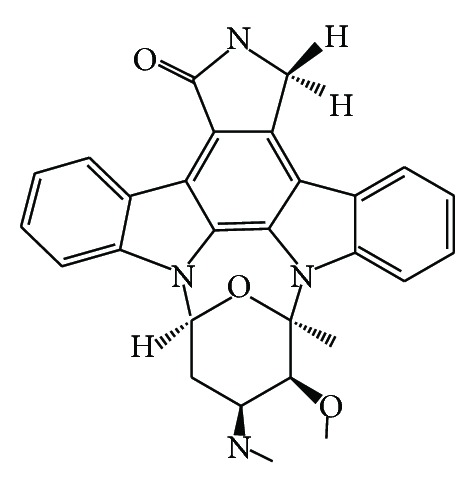
Structure of indolocarbazole alkaloid staurosporine.

The cultivation of* Streptomyces* sp. strains associated with the Mediterranean sponges* Aplysina aerophoba*,* Axinella polypoides*,* Tedania* sp., and* Tethya* sp., collected in Croatia, has yielded the indolocarbazole alkaloid staurosporine (Figure 8). The compound was screened for anti-infective activities and showed significant antiparasitic activity against* Leishmania major* with IC_50_ 5.30 *μ*M and* T. b. brucei* with IC_50_ 0.022 *μ*M [83]. Staurosporine was first isolated in 1977 from* Streptomyces staurisporeus* and later also from other actinomycetes as well as cyanobacteria. In the meantime several staurosporine analogues were isolated from actinomycetes and also from marine invertebrate, including among others sponges, mollusks, and tunicates. Interestingly, in several cases staurosporine and related derivatives were isolated from Streptomyces sp. The occurrence of such compound may suggest the presence of associated microorganisms responsible for the biosynthesis of staurosporine [84].

**Figure 9:**
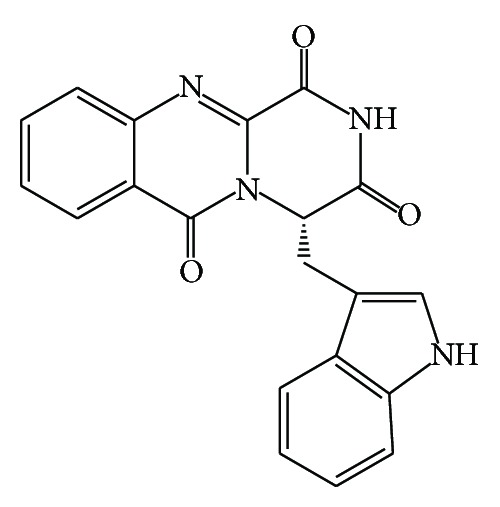
Structure of pyrazinoquinazoline-derived alkaloid oxoglyantrypine.

A novel indole alkaloid containing pyrazinoquinazoline-derivative framework, (14*S*)-oxoglyantrypine (Figure 9), isolated from the culture of the mangrove-derived fungus* Cladosporium* sp. PJX-41 exhibited activity against influenza virus H1N1 with IC_50_ value of 85 *μ*M [85].

**Figure 10:**
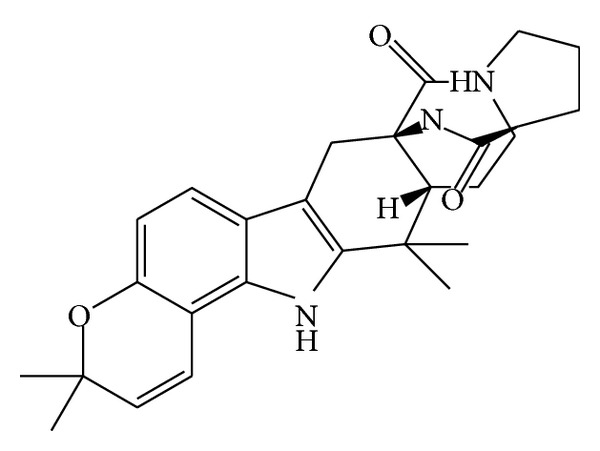
Structure of prenylated indole alkaloids (−)-stephacidin A.

From marine* Aspergillus* sp., a prenylated indole alkaloid was isolated and its antibacterial activity was assayed. (−)-Stephacidin A (Figure 10) showed MIC value of 21.5 *μ*M against* S. epidermidis *[86].

**Figure 11:**
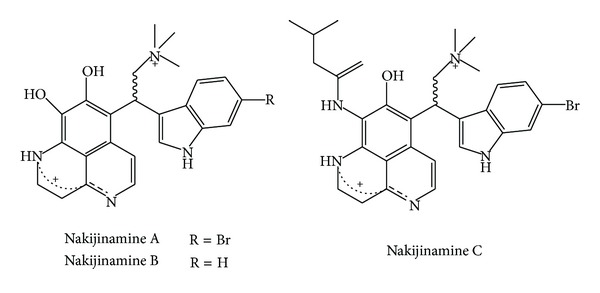
Structures of heteroaromatic aaptamine-type indole alkaloids.

Nakijinamines A, B, and C (Figure 11) have been shown to present antibacterial and antifungal activity. These compounds present a heteroaromatic aaptamine-type framework and a halogenated indole moiety. Nakijinamine A was active against* C. albicans *(IC_50_ 0.25 *μ*g/mL),* C. neoformans *(IC_50_ 0.5 *μ*g/mL),* Trichophyton mentagrophytes *IC_50_ (0.25 *μ*g/mL),* S. aureus *(MIC 16 *μ*g/mL),* B. subtilis *(MIC 16 *μ*g/mL), and* Micrococus luteus* (MIC 2 *μ*g/mL). Nakijinamines B and C were only active against* C. albicans* with IC_50_ value of 8 *μ*g/mL each [87].

**Figure 12:**
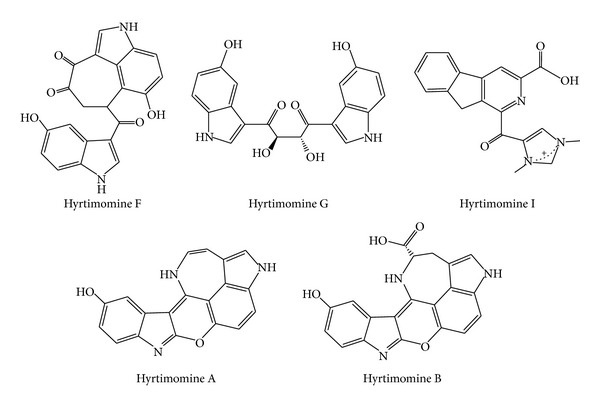
Structures of hyrtimomine-type alkaloids.

Alkaloids with hyrtimomine-type framework (Figure 12) were isolated from* Hyrtios* spp. Hyrtimomines F, G, and I exhibited inhibitory effects against* Aspergillus niger* (IC_50_ 8.0 *μ*g/mL each), while hyrtimomine I showed inhibitory effect against* C. neoformans* (IC_50_ 4.0 *μ*g/mL). Hyrtimomines A and B also showed antifungal activities against* C. albicans* (IC_50_ 1.0 *μ*g/mL each) and* C. neoformans* (IC_50_ 2.0 and 4.0 *μ*g/mL, resp.). Hyrtimomine A exhibited an inhibitory activity against* A. niger* (IC_50_ 4.0 *μ*g/mL) [88].

**Figure 13:**
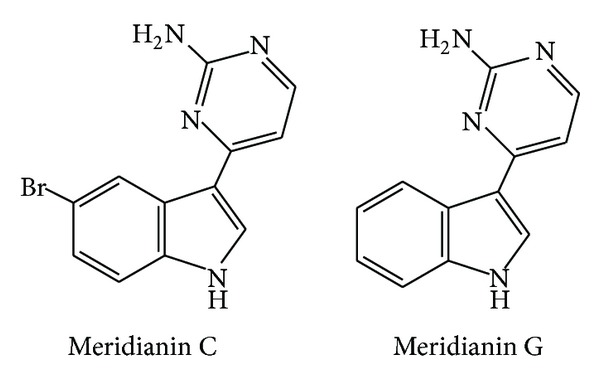
Structures of meridianins C and G.

Meridianins A-G (Figure 13) comprise indole alkaloids substituted at the C-3 position by a 2-aminopyridine ring and had been previously isolated from the tunicate* Aplidium meridianum*. Investigations on the antimalarial and antileishmanial activity of meridianin C and G were carried out and showed that these two compounds inhibited* P. falciparum* D6 and W2 clones with IC_50_ values in the range of 4.4 to 14.4 *μ*M. Meridianin C was also active against* L. donovani* promastigotes showing an IC_50_ value of 64 *μ*M [89].

Simple indole-derived alkaloids were isolated from the marine bacterium* Bacillus pumilus*. Compounds 3-hydroxyacetylindole and *N*-acetyl-*β*-oxotryptamine (Figure 3) promoted inhibition on the growth of amastigotes from* T. cruzi* with IC_50_ values of 20.6 and 19.4 *μ*M, respectively [90].

**Figure 14:**
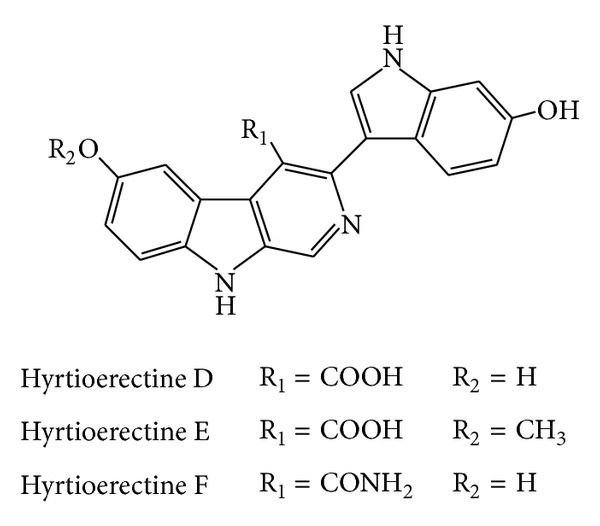
Structures of hyrtioerectines D–F.

Hyrtioerectines D–F (Figure 14) were isolated from* Hyrtios* species and had their antimicrobial activities screened. These *β*-carboline-derived compounds showed inhibition zones of 17, 9, and 14 mm against* C. albicans* compared to 35 mm illustrated by clotrimazole at the same concentration. In addition, they caused inhibition zones of 20, 10, and 16 mm against* S. aureus*, respectively, compared to 30 mm illustrated by ampicillin, as well as inhibition zones of 7–9 mm against* Pseudomonas aeruginosa* compared to 30 mm illustrated by imipenem [91].

As noted in the study above, the main source of marine indole alkaloids are sponges, whilst pharmacological assays focus on activity toward parasites from the genus* Plasmodium* and* Trypanosoma*. Although few indole alkaloids from marine sources have been tested for anti-infective activity, there are some promising compounds which demonstrate high level of activity against several infectious diseases agents including bacteria, fungi, and protozoa. These active alkaloids show simple as well as complex frameworks and unlike terrestrial organisms, they frequently contain halogenated moieties. Therefore this review highlighted some marine indole alkaloids which may be considered as potential starting points for the development of novel agents for the treatment of infectious diseases.
